# Antiviral Activities of Several Oral Traditional Chinese Medicines against Influenza Viruses

**DOI:** 10.1155/2015/367250

**Published:** 2015-10-08

**Authors:** Lin-Lin Ma, Miao Ge, Hui-Qiang Wang, Jin-Qiu Yin, Jian-Dong Jiang, Yu-Huan Li

**Affiliations:** ^1^Institute of Medicinal Biotechnology, Chinese Academy of Medical Sciences and Peking Union Medical College, Beijing 100050, China; ^2^Institute of Materia Medica, Chinese Academy of Medical Sciences and Peking Union Medical College, Beijing 100050, China

## Abstract

Influenza is still a serious threat to human health with significant morbidity and mortality. The emergence of drug-resistant influenza viruses poses a great challenge to existing antiviral drugs. Traditional Chinese medicines (TCMs) may be an alternative to overcome the challenge. Here, 10 oral proprietary Chinese medicines were selected to evaluate their anti-influenza activities. These drugs exhibit potent inhibitory effects against influenza A H1N1, influenza A H3N2, and influenza B virus. Importantly, they demonstrate potent antiviral activities against drug-resistant strains. In the study of mechanisms, we found that Xiaoqinglong mixture could increase antiviral interferon production by activating p38 MAPK, JNK/SAPK pathway, and relative nuclear transcription factors. Lastly, our studies also indicate that some of these medicines show inhibitory activities against EV71 and CVB strains. In conclusion, the 10 traditional Chinese medicines, as kind of compound combination medicines, show broad-spectrum antiviral activities, possibly also including inhibitory activities against strains resistant to available antiviral drugs.

## 1. Introduction

Influenza is an infectious disease with serious threats to human health. Influenza is estimated to result in about 3 to 5 million cases of severe illness and about 250,000 to 500,000 deaths worldwide every year [1, 2]. Currently, influenza vaccination and antiviral drugs are the primary way to prevent the disease. However, influenza vaccination may be less effective among the elderly and fails to supply protection against heterosubtype viruses [3].

To date, only three classes of antiviral drugs have been approved for use in the clinic: M2 ion-channel inhibitors (i.e., amantadine and rimantadine), neuraminidase inhibitors (i.e., oseltamivir, zanamivir, and peramivir), and RNA-dependent RNA polymerase (RdRp) inhibitor (favipiravir). M2 ion-channel inhibitors are effective only against type A virus, and their efficacies are limited because of M2 inhibitors resistance occurrence [4]. Nowadays, almost all circulating influenza A viruses that recovered from humans are resistant to adamantanes [5]. Although neuraminidase (NA) inhibitors are active against both type A and type B viruses, oseltamivir-resistance occurred in A(H1N1)2009 virus and even in A(H7N9)2013 virus [6–9]. In addition to drug-resistance, both inhibitors only worked at the early phase of virus infection. RdRp inhibitor favipiravir was only approved in Japan and its efficacy needs to be confirmed in other countries. Therefore, these highlight an urgency to develop new feasible measures to inhibit influenza virus.

TCMs are traced back to Yellow Emperor and they have served Chinese people for thousand years [10]. Patients with mild influenza symptom usually prefer taking TCMs in China. Recently, some research also verified their efficacies on the cure for influenza. One retrospective study indicated that TCM therapy was effective for reducing duration of viral shedding in H1N1 patients with body temperature over 38.0°C [11], and another randomized trial suggested that Chinese traditional therapy ma-xing-shi-gan–yin-qiao-san showed a good efficacy similar to that of oseltamivir in the treatment of H1N1 influenza [12]. However, lack of intensive studies on antiviral activities and mechanisms limits the development of TCMs in antiviral therapy.

Herein, we selected 10 commercial oral Chinese patent medicines, including Jinzhen oral liquid, Antiviral oral liquid, Compound Yuxingcao mixture, Qingre Jiedu oral liquid from two factories, Children's Qingre oral liquid, Qingkailing oral liquid, Xiaoqinglong mixture, Compound Qinlan oral liquid, and Cold liquid, which consist of several herb ingredients. We studied their anti-influenza activities* in vitro* and explored mechanisms of Xiaoqinglong mixture against influenza.

## 2. Materials and Methods

### 2.1. Drugs

10 traditional Chinese medicines, including Jinzhen oral liquid (Jiangsu Kanion Pharmaceutical Co., Ltd., lot number 130110), Antiviral oral liquid (Henan Kangxin 100 Co., Ltd., lot number 41022199), Compound Yuxingcao mixture (Zhejiang Huisong Pharmaceutical Co., Ltd., lot number 20026198), Qingre Jiedu oral liquid (Harbin Pharm. Group Sanjing Pharmaceutical Co., Ltd., lot number 13012526), Qingre Jiedu oral liquid (Beijing Tongrentang Technology Development Co., Ltd., lot number 12261629), Children's Qingre oral liquid (Beijing Tongrentang Technology Development Co., Ltd., lot number 12261652), Qingkailing oral liquid (Guangzhou Baiyun Mountain Ming Xing Pharmaceutical Co., Ltd., lot number 130107), Xiaoqinglong mixture (Hubei Newland Pharmaceutical Co., Ltd., lot number 42021012), Compound Qinlan oral liquid (Heilongjiang Zbd Pharmaceutical Co., Ltd., lot number 20120508), and Cold liquid (Beijing Tongrentang Technology Development Co., Ltd., lot number 12140978), were purchased from People Sunshine Pharmacy of Beijing. Since these 10 oral traditional Chinese medicines are all oral liquid medications, their concentrations used in this study were expressed as *μ*L/mL. Oseltamivir phosphate (NICPBP, lot number 101096-200901) and amantadine hydrochloride (Sigma, lot number 665-66-7) were used as positive control drugs. The detailed prescriptions of these drugs were displayed at Supplementary Table 1 (in Supplementary Material available online at http://dx.doi.org/10.1155/2015/367250).

### 2.2. Cells and Viruses

Madin-Darby canine kidney (MDCK) cells were maintained in minimum essential medium (MEM) containing 10% fetal bovine serum (FBS) and 1% MEM nonessential amino acids solution (NEAA). RAW264.7 cells and Vero cells were grown in Dulbecco's Modified Eagle Medium (DMEM) and MEM, respectively, supplemented with 10% FBS.

Influenza strain A/Fort Monmouth/1/1947(H1N1) was purchased from the ATCC. Influenza strains A/Wuhan/359/1995(H3N2), BV/shenzhen/155/2005, clinical isolated A/Jinnan/15/2009(H1N1), and A/Zhuhui/1222/2010(H3N2), which are resistant to oseltamivir and amantadine, respectively, were kindly donated by the Institute for Viral Disease Control and Prevention, China Centers for Disease Control and Prevention, Yuelong Shu Professor. Viral stocks of these strains were prepared by passaging them in 10-day-old embryonated chicken eggs for 2 or 3 days.

Enterovirus 71 (EV71) strain SHZH98 isolated from the throat swab sample of HFMD case occurring in 1998 in China was kindly provided by Dr. Qi Jin, Institute of Pathogen Biology, Chinese Academy of Medical Science and Peking Union Medical School, Beijing, China. EV71 strain BrCr (VR-1775) and H (VR-1432) were purchased from the ATCC. EV71 strain JS-52 was a kind gift from Dr. Xiangzhong Ye, Beijing Wantai Biological Pharmacy Enterprise Co., Ltd. EV71 strains SHZH98, BrCr, H, and JS-52 were passaged in Vero cells. Coxsackievirus B (CVB) strains CVB2 (strain Ohio-1), CVB3 (strain Nancy), CVB4 (strain J.V.B.), and CVB6 (strain Schmitt) were all obtained from the ATCC and passaged in Vero cells.

### 2.3. Drug Cytotoxicity

Effects of 10 oral medicines on viability of MDCK cells were evaluated by MTT assay. Briefly, MDCK cells grown in 96-well plate were incubated with serial twofold dilutions of oral medicines. After 72 h, 10 *μ*L 5 mg/mL MTT (Promega, San Luis Obispo, CA, USA) dissolved in phosphate-buffered saline (PBS) was added to the cell culture medium. After 4 h incubation, the medium was aspirated and replaced by 150 *μ*L of DSMO. The plates were shaken for 10 min and the absorbance was read at 450 nm on Enspire (Perkin Elmer, Waltham, MA, USA).

### 2.4. Cytopathic Effect (CPE) Assays

MDCK cells seeded in 96-well plates were washed once with PBS and then treated with diluted virus solutions at 100 TCID_50_ (50% tissue culture infective dose). Following virus adsorption for 2 h at 37°C, the unbound viruses were removed and replaced by maintenance medium with 2 *μ*g/mL TPCK-treated trypsin (Worthington, Lakewood, Colorado, USA) and 0.08% BSA (Beijing Yuan Heng Golden Horse Biological Technology Development Co., Ltd.) with or without the tested compounds. The virus-induced CPE was recorded when the CPE of virus control group reached 100%, and 50% cell-inhibitory concentrations (IC_50_) were determined using Reed and Muench method. The selectivity index (SI) values were calculated with TC_50_/IC_50_.

For anti-EV71 and anti-CVB activity assays, 100 TCID_50_ viruses were added to Vero cells grown in 96-well plates and then the unbound viruses were replaced by maintenance medium containing 2% FBS and tested drugs after 1 h virus adsorption.

### 2.5. Western Blotting

Total MDCK or RAW264.7 cellular proteins were extracted and the protein concentrations were determined by BCA Protein Assay Kit (Thermo Fisher Scientific, Waltham, MA, USA). Equal amount of proteins were subjected to SDS-PAGE and then electrotransferred to PVDF membrane (Millipore, Billerica, MA, USA). After being blocked by 5% milk, the membranes were incubated with mouse antibodies against influenza A M2, influenza A NS1, PKR, actin (Santa Cruz, Dallas, Texas, USA), rabbit antibodies against phosphospecific and total p38/JNK-SAPK/p65/c-jun (Santa Cruz), as well as influenza A PA (Gene Tex, Irvine, CA, USA). Lastly, HRP-conjugated secondary antibodies were applied and the signals were detected using ECL detection kit (GE Healthcare Life Sciences, Pittsburgh, PA, USA).

### 2.6. Quantitative Real-Time PCR

Total RNA was isolated from MDCK and RAW264.7 cells using RNeasy Mini Kit (Qiagen, Germantown, MD, USA). The viral M2 mRNA of influenza virus strain A/Fort Monmouth/1/1947, GAPDH mRNA (mouse and dog), and IFN-*α*/*β* mRNA (mouse) were amplified by quantitative real-time PCR with specific primers (Table 1). The real-time PCR was carried out using SuperScript III Platinum SYBR Green One-Step qRT-PCR kit (Invitrogen, Carlsbad, California, USA) with the following procedures: 50°C for 3 min, 95°C for 5 min, followed by 35 cycles of 95°C for 15 s, and 60°C for 30 s. The relative amounts of influenza M2 mRNA and IFN-*α*/*β* mRNA were calculated by comparative Ct method after normalizing the quantity of GAPDH.

## 3. Results

### 3.1. Anti-Influenza Activities* In Vitro*

**Figure 1:**
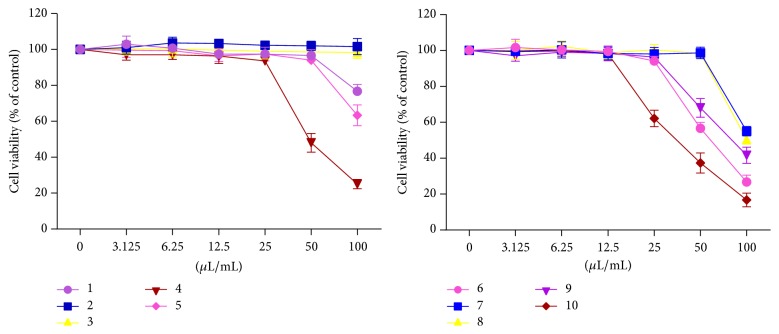
Effects of 10 oral medicines on viability of MDCK cell. Cell viability was measured by MTT; *n* = 3. The data represent the mean ± SD.

To determine the anti-influenza activities of the tested medicines, their abilities to inhibit influenza virus-induced CPE in MDCK cells were evaluated. We first determined effects of the 10 oral medicines on cell viability through MTT and the results were shown in Figure 1. Furthermore, TC_50_ and TC_0_ of these medicines were determined through the cytotoxicity assay based on CPE reduction (Table 2). The cytotoxicity of these medicines obtained from these two methods was basically consistent. According to the above results, we determined concentrations of medicines used in anti-influenza activity assay.

The data from anti-influenza activity assay demonstrated that these 10 medicines significantly inhibited A/Fort Monmouth/1/1947(H1N1), with the IC_50_ values < 10 *μ*L/mL and SI > 10 (Table 3). Importantly, almost all the tested medicines displayed significant inhibitory activities against oseltamivir-resistant A/Jinnan/15/2009(H1N1) except Jinzhen oral liquid (1) with IC_50_ of 39.82 ± 11.24 *μ*L/mL. As for H3N2 viruses, except for Qingkailing oral liquid (7) and Cold liquid (10), all the other medicines inhibited the replication of A/Wuhan/359/1995(H3N2). Of all the tested medicines, Children's Qingre oral liquid (6), Xiaoqinglong mixture (8), and Compound Qinlan oral liquid (9) exhibited strong antiviral activities against amantadine-resistant A/Zhuhui/1222/2010(H3N2), with IC_50_ values of 7.43 ± 1.44 *μ*L/mL, 2.50 ± 0.75 *μ*L/mL, and 7.52 ± 3.06 *μ*L/mL, respectively. In addition, inhibitory activities of these medicines against BV/shenzhen/155/2005 were also evaluated. Among these detected medicines, Antiviral oral liquid (2), Qingre Jiedu oral liquid (5), and Xiaoqinglong mixture (8) as well as Cold liquid (10) significantly inhibited the virus replication with their IC_50_ values lower than 10 *μ*L/mL.

As summarized in Table 3, these 10 oral proprietary Chinese medicines showed strong abilities of inhibiting at least 3 different influenza strains containing oseltamivir-resistant H1N1 and amantadine-resistant H3N2.

### 3.2. Influenza Proteins and RNA Expression Inhibition Assay

**Figure 2:**
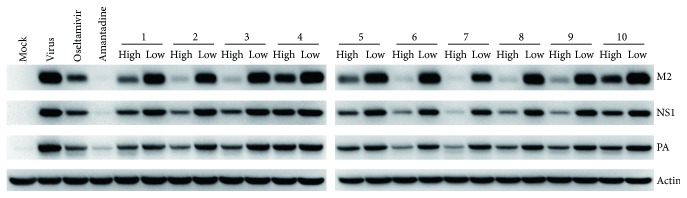
The effects of 10 medicines on influenza proteins expression.* Oseltamivir*: 10 *μ*g/mL;* amantadine*: 1 *μ*g/mL; 1–3: high concentrations of 50 *μ*L/mL, low concentrations of 10 *μ*L/mL; 4–8: high concentrations of 20 *μ*L/mL, low concentrations of 4 *μ*L/mL; 9-10: high concentrations of 10 *μ*L/mL, low concentrations of 2 *μ*L/mL; mock: normal cells without treatment; virus: cells infected with A/Fort Monmouth/1/1947 at 0.005 multiplicity of infection (MOI).

To further confirm the inhibitory action of these medicines against influenza, viral proteins expressions were evaluated by Western blotting assay. As presented in Figure 2, in which the viral protein levels were normalized by beta-actin, these 10 oral medicines markedly decreased the expression of M2, NS1, and PA proteins of A/Fort Monmouth/1/1947 in a dose-dependent manner. The tested 10 medicines at the high concentrations exhibited potent inhibitory activities on the viral proteins expression. Moreover, of the tested medicines, Antivial oral liquid (2), Compound Yuxingcao mixture (3), Children's Qingre oral liquid (6), Qingkailing oral liquid (7), Xiaoqinglong mixture (8), and Compound Qinlan oral liquid (9) at their high concentrations almost completely inhibit the expression of the viral proteins.

**Figure 3:**
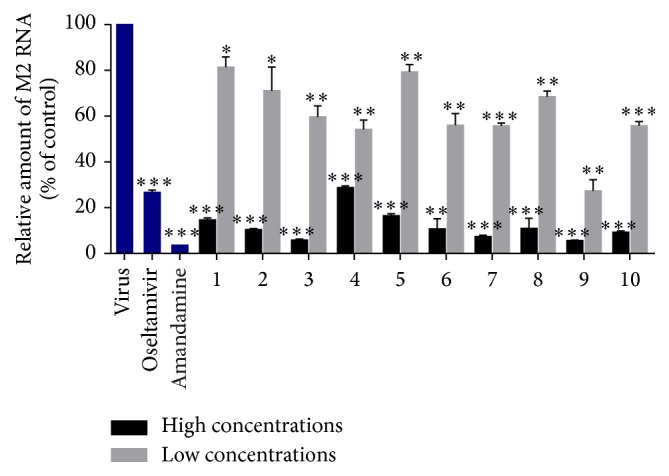
The effects of 10 medicines on influenza RNA expression.* Oseltamivir*: 10 *μ*g/mL;* amantadine*: 1 *μ*g/mL; 1–3: high concentrations of 50 *μ*L/mL, low concentrations of 10 *μ*L/mL; 4–8: high concentrations of 20 *μ*L/mL, low concentrations of 4 *μ*L/mL; 9-10: high concentrations of 10 *μ*L/mL, low concentrations of 2 *μ*L/mL; *n* = 3, and each value represents the mean ± SD; MOI = 0.005; ^*∗*^ *p* < 0.05, ^*∗∗*^ *p* < 0.01, and ^*∗∗∗*^ *p* < 0.001, compared with virus control.

In addition to Western blotting assay, the antiviral abilities of 10 medicines against influenza viruses were further confirmed by quantitative real-time PCR. Consistent with the results of Western blotting assay, these 10 medicines exhibited potent inhibitory action against RNA replication of A/Fort Monmouth/1/1947 under the high concentrations (Figure 3) and most of the 10 medicines at the high concentrations showed potent inhibitory effects with their abilities of inhibiting M2 RNA replication ranging from that of 10 *μ*g/mL oseltamivir to that of 1 *μ*g/mL amantadine.

### 3.3. Xiaoqinglong Mixture Enhanced Interferon (IFN) Response through Activating p38, JNK/SAPK MAPK Pathway and NF-*κ*B and AP-1 Nuclear Transcription Factors

Several studies reported that some TCMs regulate the immune system to resist viral infection [13–15]. Therefore, we speculated that antiviral mechanisms of the tested medicines may be connected with regulation of host immune system. Of all the tested medicines, Xiaoqinglong mixture (8) showed strong inhibitory activities and high SI against all the tested strains. Consequently, we selected Xiaoqinglong mixture to study its effects on host immune system.

**Figure 4:**
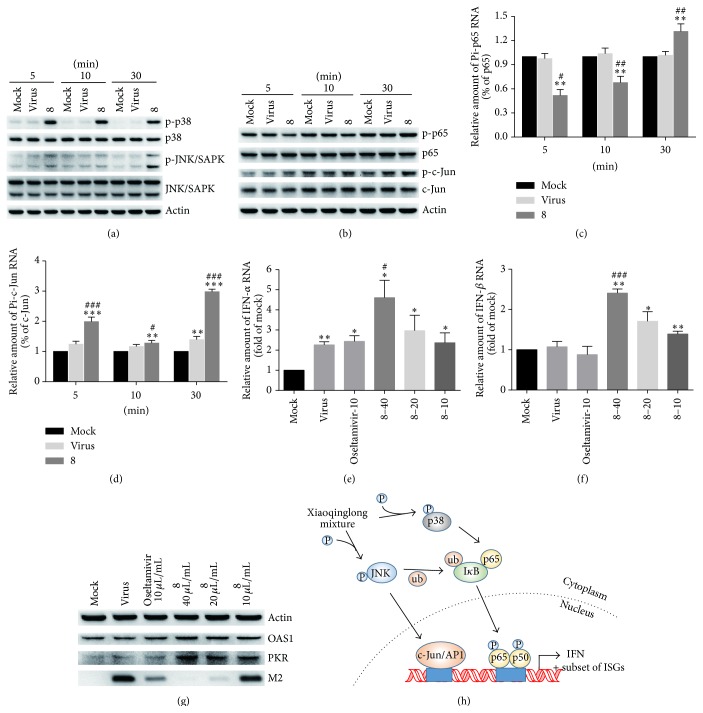
Effects of Xiaoqinglong mixture on MAPK pathway and interferon production. (a) Xiaoqinglong mixture activated p38 MAPK and phospho-JNK/SAPK pathway with concentration of 40 *μ*L/mL. (b–d) The effect of Xiaoqinglong mixture on NF-*κ*B and AP-1 nuclear transcription factor with concentration of 40 *μ*L/mL. (e-f) Xiaoqinglong mixture enhanced the RNA expression of IFN-*α* and IFN-*β* in a dose-dependent manner. Oseltamivir: 10 *μ*g/mL; 8: three diluted concentrations of 40 *μ*L/mL, 20 *μ*L/mL, and 10 *μ*L/mL, respectively. (g) Xiaoqinglong mixture enhanced the protein expression of ISGs in a dose-dependent manner. (h) Supposed schematic of Xiaoqinglong mixture activated IFN regulation system. Results are expressed as mean ± SD; ^*∗*^ *p* < 0.05, ^*∗∗*^ *p* < 0.01, and ^*∗∗∗*^ *p* < 0.001, compared with mock control; ^#^ *p* < 0.05, ^##^ *p* < 0.01, and ^###^ *p* < 0.001, compared with virus control.

To determine if Xiaoqinglong mixture could enhance host immune responses to influenza infection by inducing IFN system, the expression of IFN-*α*/*β* and IFN-stimulated genes (ISGs) was determined in RAW264.7 cells infected with A/Fort Monmouth/1/1947 and treated with 40 *μ*L/mL Xiaoqinglong mixture. The data showed that Xiaoqinglong mixture promoted the RNA replication of IFN-*α* and IFN-*β*. Furthermore, the protein levels of kinase R (PKR) and oligoadenylate synthetase 1 (OAS1), a part of ISGs, also increased in RAW264.7 in a dose-dependent manner, whereas the control drug, oseltamivir, had no effect on tested IFNs and ISGs (Figures 4(e)–4(g)).

Extensive studies have certified that p38 and c-jun N-terminal kinase (JNK) MAP kinase pathway play a critical role in activating IFN-dependent biologic effect [16–18]. To determine if Xiaoqinglong mixture enhances IFN system by regulating MAP kinase pathway, we explored the effect of Xiaoqinglong mixture on phospho-p38 MAPK and phospho-JNK/SAPK.  Figure 4(a) showed that Xiaoqinglong mixture increased phospho-p38 MAPK and phospho-JNK/SAPK protein levels at 5 min, 10 min, and 30 min. Next, we examined changes of the nuclear transcription factor NF-*κ*B and AP1 at the downstream of MAPK pathway. The results showed that Xiaoqinglong mixture increased phospho-p65 at 30 min compared with virus control group, suggesting that Xiaoqinglong mixture activated NF-*κ*B pathway. Interestingly, Xiaoqinglong mixture lowered the phospho-p65 level at 5 min and 10 min (Figures 4(b) and 4(c)). In addition, the results also indicated that Xiaoqinglong mixture increased the phospho-c-Jun protein level at 3 different time points compared with the mock control and virus control (Figures 4(b) and 4(d)).

Taken together, these results suggest that Xiaoqinglong mixture plays anti-influenza roles at least partly by activating p38 MAPK and JNK/SAPK, resulting in Type I IFN regulation system activation (Figure 4(h)).

### 3.4. Anti-Enteroviruses and Anti-Coxsackievirus Activities* In Vitro*

EV71 and CVB are both pathogenic enteroviruses with threats to human health. EV71 is one of the causative agents of hand, foot, and mouth disease in infants and children [19]. CVB may cause viral myocarditis in some cases, which can result in dilated cardiomyopathy or even death [20]. However, to date, there are no vaccines or antiviral drugs approved for preventing or treating the infection of EV71 and CVB [21, 22].

In addition to influenza virus, we also evaluated antiviral activities of these 10 oral proprietary Chinese medicines against enterovirus. Their antiviral effects were determined by CPE analysis in Vero cell. The tested EV71 strains included SZ98, H, BrCr, and JS-52, with pirodavir as a positive control. TC_50_ of drugs against Vero cell and IC_50_ against the four EV71 strains were summarized in Table 4. Of all the 10 oral liquids, Xiaoqinglong mixture (8) possessed the best inhibitory activities against EV71 strains with SI > 5. Qingkailing oral liquid (7) and Cold liquid (10) showed no antiviral effect whereas the remaining medicines had relatively weak antiviral activities against the tested strains.

The detected CVB strains involved CVB2, CVB3, CVB4, and CVB6, with ribavirin as positive control. As exhibited in Table 5, Jinzhen oral liquid (1), Compound Yuxingcao mixture (3), Qingre Jiedu oral liquid (5), Children's Qingre oral liquid (6), Compound Qinlan oral liquid (9), and Cold liquid (10) showed broad-spectrum antiviral activities against four strains, but lower than the positive control. The remaining medicines exhibited weakly inhibitory effect on part of the tested strains.

## 4. Discussion

TCMs have a long history for serving Chinese people and numerous medicines are used in the treatment of infectious disease. These ten oral traditional Chinese medicines have been approved for treatment of patients with high fever, cough, sore throat, or upper respiratory infection that resulted from viral infection. Some of them have been proved anti-influenza efficacy in animal experiments or clinic. For example, Jinzhen oral liquid has been shown effective at improving survival rate, prolonging average survival time, and alleviating lung tissue lesions in mice infected with influenza virus [23]. Antiviral oral liquid has also been reported to inhibit influenza virus proliferation and alleviate viral pulmonary lesion in mice [24]. In clinic, Jinzhen oral liquid exhibits significant efficacy and safety in children with cough, sputum, wheezing, or fever caused by acute upper respiratory tract infections [25]. Another study showed that patients that accepted Qingkailing oral liquid treatment exhibited no significant differences on duration of clinical symptoms and clinical recovery time compared with people taking oseltamivir phosphate capsules after infected H1N1 influenza [26]. However, systemic reports about the above 10 TCMs* in vitro* activities against multiple influenza virus and other viruses are scarce. Here, we evaluated a total of 10 oral proprietary Chinese medicines against influenza virus, EV71, and CVB. Of the 10 tested TCMs, 6 medicines displayed antiviral activities against influenza A H1N1, influenza A H3N2, and influenza B strains, including oseltamivir-resistant and amantadine-resistant strains, and the remaining medicines displayed antiviral abilities against at least three influenza strains. In addition, some of TCMs tested showed inhibitory activities against EV71 and CVB. The results above suggest that TCMs possess broad-spectrum antiviral activities and provide a support for continual use of clinic. Additionally, in view of their inhibitory activities against oseltamivir-resistant and amantadine-resistant strains, TCMs may be an alternative way to figure out the issues of increasing drug-resistance.

Ingredients of TCMs, which are made often from multiple plants or other materials, are very complicated. Determining effective ingredients of TCMs is of great importance to clarify their antiviral mechanisms. These 10 medicines in our study are recorded in Chinese pharmacopoeia and their detailed components are shown in Supplementary Table 1. Scutellaria baicalensis or radix isatidis is common ingredient in 9 of the 10 TCMs. Previous reports show that extracts of Scutellaria baicalensis inhibit the neuraminidase activity of influenza A virus and the fusion of the virus with endosome/lysosome [27, 28]. In addition, radix isatidis inhibited influenza virus attachment on cells by cytoprotective activity [29, 30]. Therefore, Scutellaria baicalensis or radix isatidis may be the common effective ingredients in our 10 TCMs.

In addition to direct antiviral potencies, some TCMs also exert anti-inflammatory effects to suppress the excessive inflammatory response caused by influenza infection or regulate the immune system to resist viral infection. For example, Forsythia suspensa [31], Rehmannia [32], and Gardenia [33], which are included in our studied 10 TCMs, possess anti-inflammatory properties. Also, other ingredients, such as white peony root contained in Xiaoqinglong mixture, may be involved in restoring host immunity system to exert its preventive and therapeutic effects [34]. In fact, it has been accepted that host immune responses resulting from virus infection play a vital role in clearing infection. Activating or restoring interferon (IFN) system in organism is critical for the initiation of host immune responses important to prevent virus replication [35]. Type I and Type III IFNs trigger a series of events to inhibit viral replication, resulting in the expression of ISGs such as OAS and PKR [36]. Importantly, IFN-*α* has been used for several decades in clinic and confirmed as an effective countermeasure against HBV and HCV infection. These facts suggest that new drugs activating host IFNs should be alternative or more effective countermeasures against influenza virus or other viruses, especially strains resistant to virus-targeted drugs used in clinic, given that the mutation rate of RNA virus genomes is much higher than that of host genomes.

In the present study, our results demonstrated that Xiaoqinglong mixture, a TCM, increased the production of IFN-*α*/*β* and the expression of IFN-stimulated PKR and OAS1 by activating p38 MAPK and JNK/SAPK pathway and NF-*κ*B and AP-1 nuclear transcription factor, suggesting that activation of host immune responses is an antiviral mechanism of Xiaoqinglong mixture. To our knowledge, this is the first report on anti-influenza virus mechanisms of Xiaoqinglong with direct activation of IFN system. In addition, Xiaoqinglong mixture shows inhibitory activities against EV71 and CVB. The above results also imply that Xiaoqinglong mixture may be promising in the treatment of multiple viral infected diseases including strains resistant to available drugs in view of its broad-spectrum antiviral activities and mechanisms through direct activation of IFN system.

However, it should be noted that a key question exists in TCMs studies—unclarified active ingredients, which, in fact, makes the antiviral mechanism research hard and also limits their application worldwide in the treatment of infected diseases. Therefore, the optimum combination of active components in TCMs should be the focus in future studies.

## 5. Conclusions

Taken together, as ingredient combination drugs, the 10 tested TCMs show broad-spectrum antiviral activities and their mechanisms may involve the activation of host immune responses. These results imply that TCMs may possess some advantages in preventing or treating strains resistant to drugs against single viral target. It should be said, though, that ingredients of TCMs herein discussed is not completely clear, and it is necessary to find better compound combination drugs by clarifying the components in the future study.

## Supplementary Material

The major components in each of 10 Chinese medicines are recorded in the column “prescriptions”. The data are obtained majorly from Chinese Pharmacopoeia, 2010 version.

## Figures and Tables

**Table 1:** Oligonucleotides used for real-time RT-PCR.

Oligonucleotide	Sequence (5′-3′)
5′M2	GACCRATCCTGTCACCTCTGAC
3′M2	GGGCATTYTGGACAAAKCGTCTACG
5′GAPDH (Dog)	AGTCAAGGCTGAGAACGGGAAACT
3′GAPDH (Dog)	TCCACAACATACTCAGCACCAGCA
5′GAPDH (mouse)	CTCTGGAAAGCTGTGGCGTGATG
3′GAPDH (mouse)	ATGCCAGTGAGCTTCCCGTTCAG
5′IFN-*α* (mouse)	CCTGTGTGATGCAACAGGTC
3′IFN-*α* (mouse)	TCACTCCTCCTTGCTCAATC
5′IFN-*β* (mouse)	AGCTCCAAGAAAGGACGAACAT
3′IFN-*β* (mouse)	GCCCTGTAGGTGAGGTTGATCT

**Table 2:** Cytotoxicities of 10 oral medicines on MDCK cells.

Drug	TC_50_	TC_0_
(1) Jinzhen oral liquid^a^	>100.00 ± 0	50.00 ± 0
(2) Antiviral oral liquid^a^	>100.00 ± 0	>100.00 ± 0
(3) Compound Yuxingcao mixture^a^	>100.00 ± 0	>100.00 ± 0
(4) Qingre Jiedu oral liquid (Sanjing)^a^	48.07 ± 10.02	25.00 ± 0
(5) Qingre Jiedu oral liquid (Tongrentang)^a^	>100.00 ± 0	50.00 ± 0
(6) Children's Qingre oral liquid^a^	57.74 ± 10.23	25.00 ± 0
(7) Qingkailing oral liquid^a^	100.00 ± 15.67	50.00 ± 0
(8) Xiaoqinglong mixture^a^	100.00 ± 0	50.00 ± 0
(9) Compound Qinlan oral liquid^a^	80.00 ± 0	25.00 ± 0
(10) Cold liquid^a^	33.00 ± 2.00	12.50 ± 0
Oseltamivir^b^	>200.00 ± 0	>200.00 ± 0
Amantadine^b^	39.22 ± 5.64	20.00 ± 0

Note: ^a^ *μ*L/mL; ^b^ *μ*g/mL.

TC_50_: 50% toxicity concentration.

TC_0_: the maximal nontoxic concentration.

**Table 3:** Activities of 10 oral medicines against 5 influenza strains.

Drug	A/Fort Monmouth/1/1947		A/Jinnan/15/2009		A/Wuhan/359/1995		A/Zhuhui/1222/2010		BV/shenzhen/155/2005	
	IC_50_	SI	IC_50_	SI	IC_50_	SI	IC_50_	SI	IC_50_	SI
1^a^	8.78 ± 8.17	>11.39	39.82 ± 11.24	>2.51	2.57 ± 0.09	>38.91	32.60 ± 14.64	>3.07	67.68 ± 14.05	>1.48
2^a^	4.27 ± 3.14	>23.42	2.42 ± 1.13	>41.32	12.8 ± 19.5	>7.81	21.06 ± 17.36	>4.75	6.70 ± 3.25	>14.93
3^a^	9.49 ± 4.89	>10.54	6.64 ± 0.68	>15.06	12.5 ± 16.6	>8.00	23.11 ± 0	>4.33	11.11 ± 0	>9.00
4^a^	3.12 ± 1.92	15.41	7.37 ± 4.58	6.52	5.33 ± 2.90	9.02	21.18 ± 2.74	2.27	>25.00	—
5^a^	2.21 ± 0	>45.25	5.82 ± 3.61	>17.18	9.32 ± 11.82	>10.73	19.24 ± 0	>5.20	8.57 ± 0.18	>11.67
6^a^	2.81 ± 1.59	20.55	6.52 ± 1.15	8.86	1.19 ± 0.18	48.52	7.43 ± 1.44	7.77	11.11 ± 0	5.20
7^a^	2.24 ± 0.60	44.64	5.30 ± 1.28	18.87	>50.00	—	15.23 ± 1.29	6.57	16.70 ± 2.11	5.99
8^a^	3.77 ± 0.21	26.53	2.79 ± 1.04	35.84	8.36 ± 6.56	11.96	2.50 ± 0.75	40	5.11 ± 3.25	19.57
9^a^	0.88 ± 0.26	90.91	2.48 ± 0	32.26	1.45 ± 0.17	55.17	7.52 ± 3.06	10.64	>11.11	—
10^a^	2.88 ± 1.82	11.46	3.13 ± 1.83	10.54	>12.50	—	7.06 ± 0.91	4.67	2.87 ± 0	11.50
Oseltamivir^b^	1.01 ± 0.40	>198.02	>200.00	—	0.94 ± 0.32	>212.77	1.29 ± 0.25	>155.04	91.07 ± 34.51	>2.20
Amantadine^b^	0.17 ± 0.06	230.71	2.86 ± 0.91	13.71	2.86 ± 0.78	13.71	5.93 ± 0.27	6.61	>20	—

Note: ^a^ *μ*L/mL; ^b^ *μ*g/mL.

IC_50_: 50% cell-inhibitory concentrations; SI: selectivity index; SI = TC_50_/IC_50_.

“—”: no antiviral activity at the maximal nontoxic concentration.

*n* = 3: each value represents the mean ± SD.

**Table 4:** Activities of 10 oral proprietary Chinese medicines against 4 enterovirus 71 strains.

Drug	TC_50_	SZ98		H		BrCr		JS52	
		IC_50_	SI	IC_50_	SI	IC_50_	SI	IC_50_	SI
1^a^	62.06 ± 7.49	21.34 ± 5.18	2.91	16.9 ± 6.22	3.67	9.37 ± 4.12	6.62	16 ± 7.48	3.88
2^a^	66.9 ± 15.87	>25	—	23.15 ± 2.62	2.89	13.26 ± 6.25	5.05	19.84 ± 7.3	3.37
3^a^	36.37 ± 20.88	21.34 ± 5.18	1.7	18.75 ± 8.84	1.94	10.66 ± 9.92	3.41	12.3 ± 4.89	2.96
4^a^	36.24 ± 3.44	19.49 ± 2.56	1.86	10.48 ± 2.86	3.46	5.48 ± 2.56	6.61	10.48 ± 2.86	3.46
5^a^	6.51 ± 2.02	5.79 ± 0.65	1.12	5.34 ± 1.29	1.22	4.23 ± 2.85	1.54	4.52 ± 2.45	1.44
6^a^	5.03 ± 0.53	2.67 ± 0.65	1.88	2.48 ± 0.91	2.03	1.86 ± 0.87	2.7	1.84	2.73
7^a^	6.9 ± 0.84	>3.13	—	>3.13	—	>3.13	—	>3.13	—
8^a^	17.65 ± 1.62	3.36 ± 2.79	5.25	2.77 ± 1.34	6.37	2.48 ± 1.75	7.12	2.31 ± 1.41	7.64
9^a^	3.42 ± 1.17	2.35 ± 1.1	1.46	2.12 ± 0.78	1.61	1.83 ± 1.18	1.87	1.64 ± 0.81	2.09
10^a^	6.79 ± 1.67	>3.7	—	>3.7	—	>3.7	—	>3.7	—
Pirodavir^b^	12.02	0.1 ± 0.1	120.2	0.15 ± 0.04	80.13	0.06 ± 0	200.33	0.06 ± 0.05	200.33

Note: ^a^ *μ*L/mL; ^b^ *μ*g/mL; *n* = 3: each value represents the mean ± SD.

**Table 5:** Activities of 10 oral proprietary Chinese medicines against 4 coxsackievirus B strains.

Drug	TC_50_	CVB2		CVB3		CVB4		CVB6	
		IC_50_	SI	IC_50_	SI	IC_50_	SI	IC_50_	SI
1^a^	62.06 ± 7.49	1.96 ± 0.98	31.66	6.14 ± 3.33	10.11	6.14 ± 3.33	10.11	13.39 ± 6.62	4.63
2^a^	66.9 ± 15.87	>33.33	—	6.84 ± 2.83	9.78	15.96 ± 2.84	4.19	7.13 ± 0.99	9.38
3^a^	36.37 ± 20.88	3.89 ± 1.52	9.35	10.56 ± 6.1	3.44	2.2 ± 1.7	16.53	6.29 ± 1.47	5.78
4^a^	36.24 ± 3.44	>11.11	—	4.19 ± 1.91	8.65	6.37 ± 2.31	5.69	7.7 ± 0	4.71
5^a^	6.51 ± 2.02	1.15 ± 0.56	5.66	1.98 ± 0.21	3.29	2.24 ± 0.57	2.91	2.43 ± 0.25	2.68
6^a^	5.03 ± 0.53	0.68 ± 0.16	7.40	2.27 ± 0.74	2.22	0.74 ± 0.18	6.8	0.98 ± 0.22	5.13
7^a^	6.9 ± 0.84	1.74 ± 1.08	3.97	>3.13	—	3.7 ± 0	1.86	>3.7	—
8^a^	17.65 ± 1.62	0.94 ± 0.29	18.78	3.22 ± 1.98	5.48	>3.7	—	>3.7	—
9^a^	3.42 ± 1.17	0.45 ± 0.07	7.60	1.5 ± 1.01	2.28	0.64 ± 0.04	5.34	1.1 ± 0.22	3.11
10^a^	6.79 ± 1.67	1.1 ± 0.6	6.17	1.77 ± 0.32	3.84	2.24 ± 0.57	3.03	1.77 ± 0.32	3.84
RBV^b^	>10000	554 ± 37	>18.05	366 ± 104	>27.32	331 ± 109	>30.21	336 ± 74	>29.76

Note: ^a^ *μ*L/mL; ^b^ *μ*g/mL; *n* = 3: each value represents the mean ± SD.
