# Supplementary material for: Antiviral Activities of Several Oral Traditional Chinese Medicines against Influenza Viruses
Source: Evid Based Complement Alternat Med. 2015 Oct 8;2015:367250. doi: 10.1155/2015/367250 (PMC4618326; doi:10.1155/2015/367250)
Supplement: Supplementary file 1 — The major components in each of 10 Chinese medicines are recorded in the column “prescriptions”. The data are obtained majorly from Chinese Pharmacopoeia, 2010 version. [file 367250.f1.pdf]

Supplementary Table 1. The detail prescriptions of 10 oral medicines

**Drug**	**prescriptions**
1.Jinzhen oral liquid	antelope horn, fritillary bulb, rhubarb, scutellaria baicalensis, lapis chloriti, gypsum fibrosum, calculus bovis factitius, liquorice
2.Antiviral oral liquid	radix isatidis, gypsum fibrosum, reed rhizome, rehmannia, radix curcumae, rhizoma anemarrhenae, acorus tatarinowii, pogostemon cablin, forsythia suspensa
3.Compond yuxingcao mixture	herba houttuyniae, scutellaria baicalensis, radix isatidis, forsythia suspensa, honeysuckle
4-5. Qingre Jiedu oral liquid	gypsum fibrosum, honeysuckle, scrophularia ningpoensis, rehmanniae, forsythia suspensa，gardenia，manyflower gueldenstaedtid herb，scutellaria baicalensis, gentian, radix isatidis, rhizoma anemarrhenae, radix ophiopogonis
6.Children's Qingre heat oral liquid	honeysuckle, periostracum cicada, gypsum fibrosum, talc, scutellaria baicalensis, rhubarb, radix paeoniae rubra, radix isatidis, pogostemon cablin, sliced antelope horn
7. Qingkailing oral liquid	cholalic acid, nacre, hyodeoxycholic acid, gardenia, cornu bubali, radix isatidis, baicalin, honeysuckle
8. Xiaoqinglong mixture	ephedra, cassia twig, white peony root, dried ginger, asarum, prepared radix licorice, rhizoma pinellinae praeparata, schisandra chinensis
9. Compound Qinlan oral liquid	honeysuckle, scutellaria baicalensis, forsythia suspensa, radix isatidis
10. Cold liquid	notopterygium, ephedra, cassia twig, schizonepetaspike, saposhnikovia divaricata, radix angelicae dahuricae, rhizoma chuanxiong, acorus tatarinowii, the root of kudzu vine, mint, semen armeniacae amarum, angelica sinensis, scutellaria baicalensis, platycodon grandiflorum
